# Spinal subarachnoid hematoma in a woman with HELLP syndrome: a case report

**DOI:** 10.1186/1752-1947-6-152

**Published:** 2012-06-13

**Authors:** Hisako Fujimaki, Toshiyuki Nakazawa, Masaki Ueno, Takayuki Imura, Wataru Saito, Naonobu Takahira, Masashi Takaso

**Affiliations:** 1 Department of Orthopedic Surgery, Kitasato University School of Medicine, 1-15-1 Kitasato, Sagamihara, Kanagawa, 228-8555, Japan

## Abstract

**Introduction:**

Subarachnoid hemorrhages of spinal origin are extremely rare during pregnancy. We present the case of a patient with hemolytic anemia, elevated liver enzymes and low platelet count (the so-called HELLP syndrome), a potentially life-threatening complication associated with pre-eclampsia, who presented with an idiopathic spinal subarachnoid hematoma.

**Case presentation:**

At 29 gestational weeks, a 35-year-old Japanese woman was diagnosed with HELLP syndrome based on bilateral leg paralysis, diminished sensation and reflexes, and laboratory findings. The pregnancy was immediately brought to an end by Cesarean delivery. Post-operatively, an MRI scan revealed a space-occupying lesion in her thoracic spinal canal. Emergency decompression was followed by total laminectomy. A subarachnoid hematoma, partially extending as far as the ventral side, was removed. After thorough washing and drain placement, the operation was completed with the suturing of artificial dura mater. Eight months post-operatively, her lower extremity sensation had improved to a score of 8 out of 10, but improvements in her muscular strength were limited to slight gains in her toes. MRI scans taken two months post-operatively revealed edematous spinal cord changes within her medulla.

**Conclusions:**

A subarachnoid hematoma during pregnancy is extremely rare, possibly due to increased coagulability during pregnancy. However, this complication is potentially devastating should a clot compress the spinal cord or cauda equina. While several causes of hematoma have been proposed, we speculate that the factors underlying hemorrhagic diathesis in our case were the decreased platelet count characteristic of HELLP syndrome and vascular fragility due to elevated estrogen levels, in addition to increased abdominal pressure during pregnancy and pressure from the gravid uterus resulting in ruptured vessels around the spinal cord. In cases displaying a progressive lesion and severe neurological signs, prompt decompression is crucial.

## Introduction

A subarachnoid hemorrhage of spinal origin is an unusual event, accounting for only 0.05% to 1.5% of all cases with subarachnoid hemorrhage [1]. Spinal subarachnoid hematomas in pregnant women appear to be extremely rare, and to the best of our knowledge no previous reports have described idiopathic cases. We describe here a case of spinal subarachnoid hematoma associated with hemolytic anemia, elevated liver enzymes and low platelet count (the so-called HELLP syndrome), a potentially life-threatening obstetric complication.

## Case presentation

A 35-year-old Japanese woman presented to our facility with low back pain and weakness of her lower extremities. Her past history was non-contributory. No previous abnormalities had been noted during the course of her pregnancy. Her low back pain occurred without any immediate cause in gestational week 29. When weakness of her lower extremities occurred on day three of gestational week 29, causing difficulties with walking and urination, our patient consulted a local orthopedist. Blood tests revealed impaired liver function and a low platelet count, so she was brought to our emergency center.

On her initial examination, tactile and pain sensations were both reduced to 2 out of 10 (sensation graded on a 10-point scale) from the mid-thoracic level down. Manual muscle testing (MMT) for muscles below the level of the iliopsoas showed a marked decrease in strength, with a Medical Research Council scale score of 1. Her deep tendon reflexes were slightly diminished in both extremities. Complete urinary retention was noted and rectal sphincter tone was flaccid. According to the Standard Neurological Classification of Spinal Cord Injury developed by the American Spinal Injury Association (ASIA) and the International Spinal Cord Society (ISCoS), this case was defined as grade B. On hematological testing, her platelet count was markedly reduced to 1.7 × 10^4^ cells/μL. Liver function impairment was also detected (glutamic-oxaloacetic transaminase, 537U/L; glutamic-pyruvic transaminase, 294U/L; total bilirubin, 8.1 mg), while her lactate dehydrogenase and cytokeratin levels were elevated to 3766U/L and 1206U/L, respectively.

**Figure 1:**
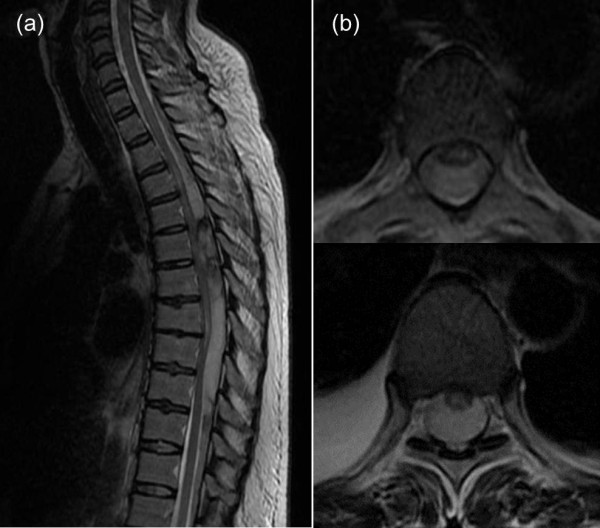
**T2-weighted thoracolumbar MRI scan images obtained immediately after Cesarean section. (a)** Sagittal plane. **(b)** Coronal plane. A space-occupying lesion showing signal hyperintensity is apparent in the upper spinal canal at the level of the second to ninth thoracic vertebrae.

After admission, HELLP syndrome was diagnosed in the obstetrics department based on the above laboratory test findings [2]. An emergency Cesarean section was successfully performed to bring the pregnancy to an end on the same day. Post-operatively, an MRI scan was performed to assess her bilateral lower limb paralysis (Figure 1). Because a space-occupying lesion was detected in her thoracic spinal canal, she was referred to our department. The lesion was diagnosed as an epidural hematoma based on the MRI findings at this time, and emergency decompression was performed the same day.

**Figure 2:**
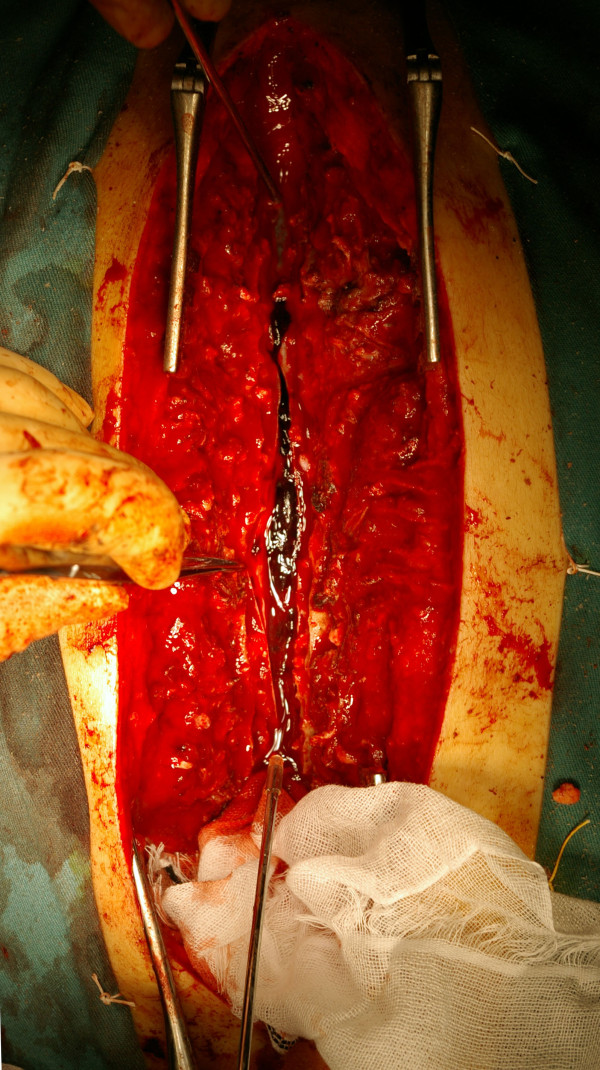
**Intra-operative image.** The upper side represents the rostral end. On incision of the dura and arachnoid mater, a subarachnoid hematoma was confirmed.

Hemilaminectomy was performed for the second to ninth thoracic vertebrae, but the epidural hematoma could not be confirmed, so the procedure was switched to a total laminectomy to allow full assessment. The dura mater was tense and swollen, and the intradural hematoma could be seen through the transparent dura. A subarachnoid hematoma was confirmed on incision of the dura and arachnoid mater, with part of the hematoma extending as far as the ventral side (Figure 2). The hematoma was removed and, after thorough washing, a drain was placed. The operation was completed with suturing of artificial dura mater.

**Figure 3:**
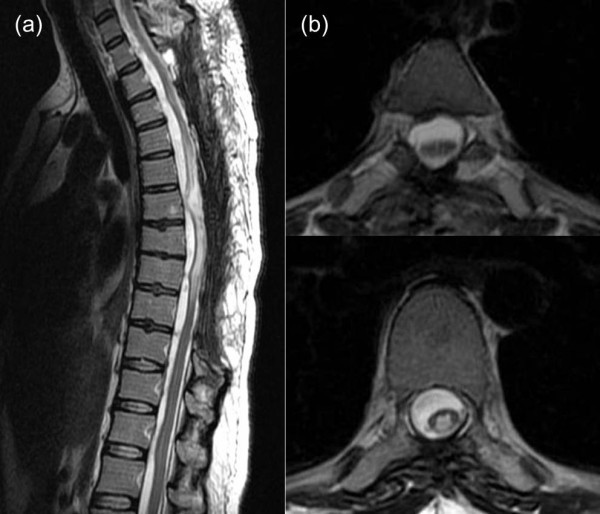
**T2-weighted thoracolumbar MRI scan images obtained two months post-operatively. (a)** Sagittal plane. **(b)** Coronal plane. The images show signal hyperintensity in the medulla and edematous changes in the spinal cord.

Because pulmonary edema was also present, the drain was withdrawn on the third post-operative day. Use of a wheelchair became possible on post-operative day 10. After eight months, her lower extremity sensation had improved to a score of 8 out of 10, although her muscular strength remained limited to a slight improvement to MMT 3 in her bilateral tibialis anterior, and extensor hallucis longus and extensor digitorum longus muscles. The ASIA/ISCoS classification was grade C. MRI performed two months post-operatively revealed signal hyperintensity within her spinal cord, suggestive of myelomalacia (Figure 3).

## Discussion

A spinal subarachnoid hematoma is much less common than either epidural or subdural hematomas. A subarachnoid hemorrhage forming a clot in the subarachnoid space is extremely rare, because the flow of cerebrospinal fluid (CSF) tends to dilute and wash away small amounts of bleeding. The subarachnoid hematoma (hemorrhage) may be primary, with a hemorrhagic source within the spinal canal, or secondary due to blood flowing from the cranial to the spinal cavity. According to Walton, primary subarachnoid hemorrhage represents approximately 1.5% of all subarachnoid hemorrhage cases [3]. Halpern *et al*. reported a spinal origin for 0.6% of their subarachnoid hemorrhage cases [4], while Sahs *et al*., in a co-operative study of a series of 6368 subarachnoid hemorrhages, reported a much lower incidence of spinal subarachnoid hemorrhage (0.05%) [5]. Furthermore, hemorrhagic disorders such as hematoma were originally believed to not develop easily during pregnancy because blood coagulability is increased in pregnant women [6]. Even a subdural hematoma developing during pregnancy, as in our case, is so rare that only two other cases have been documented, reported by Nishiyama *et al*. [7] and Tanaka *et al.*[8]. To the best of our knowledge, the present report represents the first description of an idiopathic subarachnoid hematoma occurring during pregnancy. Despite the rarity, clot formation is of considerable importance should the clot compress the spinal cord or cauda equina.

The causes of spinal subarachnoid hematoma can be roughly divided into idiopathic and secondary. Secondary causes reported to date include trauma, lumbar puncture, vascular malformation, blood dyscrasia and the use of anticoagulants. Although some etiological factors in previously idiopathic cases have been identified, the underlying mechanisms and origin of bleeding in the formation of spinal subarachnoid hematomas are not completely understood. Domenicucci *et al*. reviewed 69 cases of spinal subarachnoid hematoma. The condition was found to be associated with lumbar puncture (44.9%), coagulopathy (40.5%) and traumatic injuries (15.9%) [9]. Some authors have suggested that the rupture of arteries and radicular veins is responsible for bleeding into the CSF [10,11]. HELLP syndrome is one of the various manifestations of pre-eclampsia, and co-exists in 5% to 10% of cases [12]. This syndrome is associated with platelet depletion and disseminated intravascular coagulation. Therefore, copious bleeding into the subarachnoid space at levels in excess of the flow of CSF has been postulated to lead to hematoma formation, while the presence of coagulopathy may promote the formation of a larger hematoma. Furthermore, a study by Manalo-Estrella and Barker compared histological segments of the abdominal aorta in pregnant and non-pregnant patients [13]. They suggested that arterial and venous vessels may undergo structural changes during pregnancy, including arterial degeneration. Estrogen and progesterone excesses during the third trimester of pregnancy have been implicated as potential causes of such degenerative changes [14]. In the present case, we speculate that hemorrhagic diathesis due to the decreased platelet count characteristic of HELLP syndrome and vascular fragility [10] due to elevated estrogen levels during pregnancy were the underlying causes, in addition to the increased abdominal pressure in pregnancy and pressure from the gravid uterus resulting in ruptured vessels around the spinal cord.

A spinal subarachnoid hematoma may be treated conservatively if neurological symptoms are mild and signs of early recovery are detected. Most hematomas forming on the ventral side are not accompanied by neurological symptoms and are managed conservatively, while some on the dorsal side reportedly require decompression by percutaneous drainage [15]. Successful conservative treatment of spinal subarachnoid hematoma has been described for patients with mild neurological manifestations. Komiyama *et al*. suggested that a spinal subarachnoid hematoma anterior to the spinal cord can be treated medically, due to the lower risk of spinal compression [16]. During conservative treatment, frequent neurological examinations and MRI are recommended to detect any potential deterioration of neurological function requiring surgical intervention. However, spinal subarachnoid hematomas usually necessitate surgical decompression if neurological symptoms are severe or acute exacerbation occurs [1]. In cases such as ours, with a progressive lesion and severe neurological signs, decompression needs to be performed as soon as possible.

In the present case, because the pregnancy and HELLP syndrome were regarded as the chief problems, diagnosis of the cause of symptoms affecting the lower extremities was delayed. HELLP syndrome is defined as the presence of hemolysis, elevated liver enzymes and/or a low platelet count from the midterm of pregnancy through the early puerperal period [2]. The underlying mechanism is assumed to be microcirculatory disturbances involving the reticuloendothelial system and disseminated intravascular coagulation, although the precise pathophysiology has yet to be clarified. As a rule, the pregnancy must be terminated.

Hemorrhagic diathesis appeared to improve in our patient with delivery of the fetus. Furthermore, the influence on vessels around her spinal cord was diminished by relief of abdominal tension. Decompression was thus performed at about 36 hours after the appearance of clinical symptoms and 18 hours after detection of her lower extremity weakness, but the only strength improvement (to MMT 3) was in tibialis anterior and extensor hallucis longus and extensor digitorum longus muscles. The chances of a complete recovery improve the lower the severity of neurological signs and symptoms before treatment, and the more quickly surgical decompression can be performed. However, both mother and baby were saved in this case, and complete paralysis and hypesthesia were avoided. Since HELLP syndrome was retrospectively presumed to be a factor contributing to the development of the subarachnoid hematoma, performing the Cesarean section first appears to have been appropriate.

## Conclusions

A subarachnoid hematoma during pregnancy is extremely rare, probably due to increased coagulability. However, clinicians need to be able to recognize the symptoms and signs of a spinal subarachnoid hematoma promptly, to avoid delays in treatment and severe neurological deficits.

## Consent

Written informed consent was obtained from the patient for publication of this case report and any accompanying images. A copy of the written consent is available for review by the Editor-in-Chief of this journal.

## Competing interests

The authors declare that they have no competing interests.

## Authors’ contributions

Surgical decompression was performed by HF, TN, TI and WS. HF was a major contributor in writing the manuscript. MU participated sufficiently in the intellectual content, the analysis of data and the writing of the manuscript to take public responsibility for it. NT and MT have reviewed the manuscript, believe it represents valid work, and approved it for submission. All authors read and approved the final version of the manuscript.
